# Supplementary material for: Saccharomyces boulardii (CNCM I-745) improves intestinal damage in sepsis by remodeling intestinal flora structure
Source: Front Cell Infect Microbiol. 2025 Jul 31;15:1602792. doi: 10.3389/fcimb.2025.1602792 (PMC12350329; doi:10.3389/fcimb.2025.1602792)
Supplement: Supplementary file 1 [file DataSheet1.zip › summary/summary/7_Differential_analysis/2_LEfSe_result/Group_DW4_vs_BW4_0.05_3.0/LEfSe_Group_DW4_vs_BW4.cladogram.pdf]

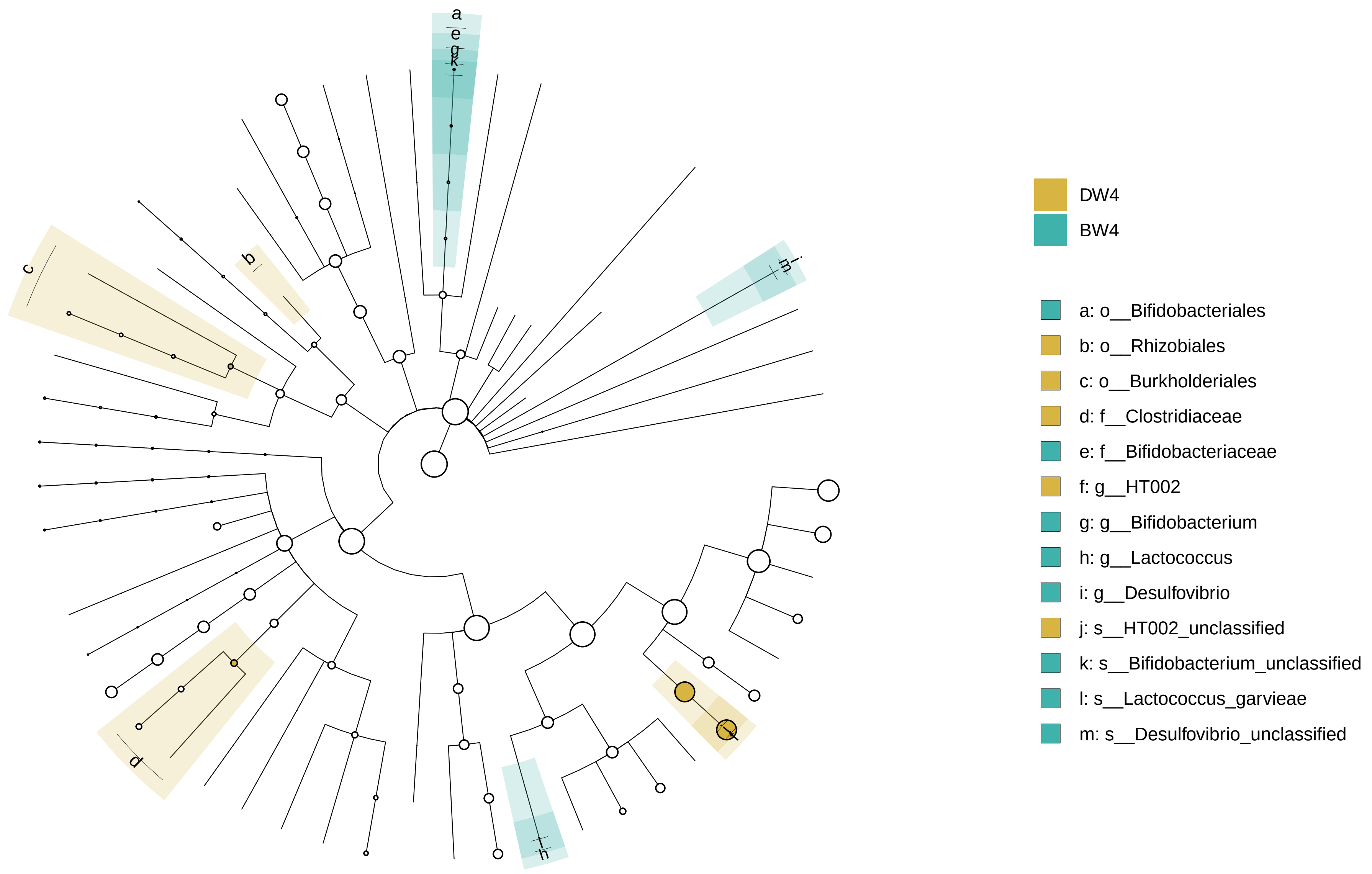
DW4
BW4
a: o__Bifidobacteriales
b: o__Rhizobiales
c: o__Burkholderiales
d: f__Clostridiaceae
e: f__Bifidobacteriaceae
f: g__HT002
g: g__Bifidobacterium
h: g__Lactococcus
i: g__Desulfovibrio
j: s__HT002_unclassified
k: s__Bifidobacterium_unclassified
l: s__Lactococcus_garvieae
m: s__Desulfovibrio_unclassified
